# Downregulation of circFASTKD1 ameliorates myocardial infarction by promoting angiogenesis

**DOI:** 10.18632/aging.202305

**Published:** 2020-12-19

**Authors:** Wen-Qing Gao, Xiao-Min Hu, Qiang Zhang, Lan Yang, Xin-Ze Lv, Shuang Chen, Peng Wu, Da-Wei Duan, Yu-Heng Lang, Meng Ning, Ke-Guan Lai, Zhi-Yuan Zhang, Bin Liang, Jing-Yu Bao, Hai-Dong Wu, Tong Li

**Affiliations:** 1 The Third Central Hospital of Tianjin, Tianjin, China; 2 Tianjin Key Laboratory of Extracorporeal Life Support for Critical Diseases, Tianjin, China; 3 Artificial Cell Engineering Technology Research Center, Tianjin, China; 4 Tianjin Key Laboratory of Early Druggability Evaluation of Innovative Drugs and Tianjin Key Laboratory of Molecular Drug Research, Tianjin International Joint Academy of Biomedicine, Tianjin, China; 5 Good Laboratory Practice Center, Tianjin International Joint Academy of Biomedicine, Tianjin, China

**Keywords:** circular RNAs, circFASTKD1, vascular endothelial cells, angiogenesis, myocardial infarction

## Abstract

Circular RNAs (circRNAs), a novel class of endogenous long non-coding RNAs, have attracted considerable attention due to their closed continuous loop structure and potential clinical value. In this study, we investigated the function of circFASTKD1 in vascular endothelial cells. CircFASTKD1 bound directly to miR-106a and relieved its inhibition of Large Tumor Suppressor Kinases 1 and 2, thereby suppressing the Yes-Associated Protein signaling pathway. Under both normal and hypoxic conditions, the ectopic expression of circFASTKD1 reduced the viability, migration, mobility and tube formation of vascular endothelial cells, whereas the downregulation of circFASTKD1 induced angiogenesis by promoting these processes. Moreover, downregulation of circFASTKD1 in mice improved cardiac function and repair after myocardial infarction. These findings indicate that circFASTKD1 is a potent inhibitor of angiogenesis after myocardial infarction and that silencing circFASTKD1 exerts therapeutic effects during hypoxia by stimulating angiogenesis *in vitro* and *in vivo*.

## INTRODUCTION

The vascular endothelium is a dynamic tissue consisting of a single layer of cells lining the inner walls of blood vessels [1]. The vascular endothelium maintains vascular tension and forms a barrier that controls the migration of various substances between the blood and various tissues, thus protecting against atherosclerosis [2–4]. Endothelial dysfunction is the key reason for the progress of all types of cardiovascular diseases.

Cardiovascular disease accounts for 45% of all non-communicable diseases and 31.5% of all deaths, and thus is the most common cause of death worldwide. The mortality rate due to cardiovascular disease is more than twice that due to cancer [5–7]. Myocardial infarction and heart failure are major cardiac syndromes, and are the main instigators of cardiovascular disease-dependent death [8, 9]. However, little is known about the molecular mechanisms whereby cardiovascular diseases develop [10, 11]. Therefore, it is very important to study the processes underlying myocardial injury and repair, and to develop treatments that effectively promote repair after myocardial infarction.

Circular RNAs (circRNAs) are non-coding RNAs that originate from exons, introns and intergenic regions [12, 13]. CircRNAs are characterized by covalently closed continuous rings that do not have 5' caps, 3' poly(A) tails or 5'-to-3' polarity. CircRNAs are usually stable, abundant, conservative and specific to cell or tissue developmental stages [14, 15]. CircRNAs are involved in numerous biological processes, including transcription, RNA splicing, RNA decay and translation. Recent studies have demonstrated that circRNA dysfunction contributes to cell dysfunction and human disease [16, 17]. CircRNAs may influence the risk of developing atherosclerotic vascular disease, liver cancer, esophageal squamous cell carcinoma, bladder cancer and other cancers [18]. Moreover, circRNAs can function as microRNA (miRNA) sponges and bind to RNA-binding proteins to alter gene expression [17, 19].

In this study, we investigated the effects of circFASTKD1 expression on the viability, migration, mobility and tube formation of vascular endothelial cells under normal and hypoxic conditions. Then, we assessed whether circFASTKD1 functioned as a competing endogenous RNA for miRNAs, and examined the signaling pathways impacted by its activity. Finally, we analyzed whether downregulating circFASTKD1 in the hearts of mice could improve cardiac function and repair after myocardial infarction. Our study has revealed a potential strategy for revascularization, and has important implications for new therapeutic approaches for myocardial infarction.

## RESULTS

### CircFASTKD1 suppresses angiogenesis in vascular endothelial cells

**Figure 1:**
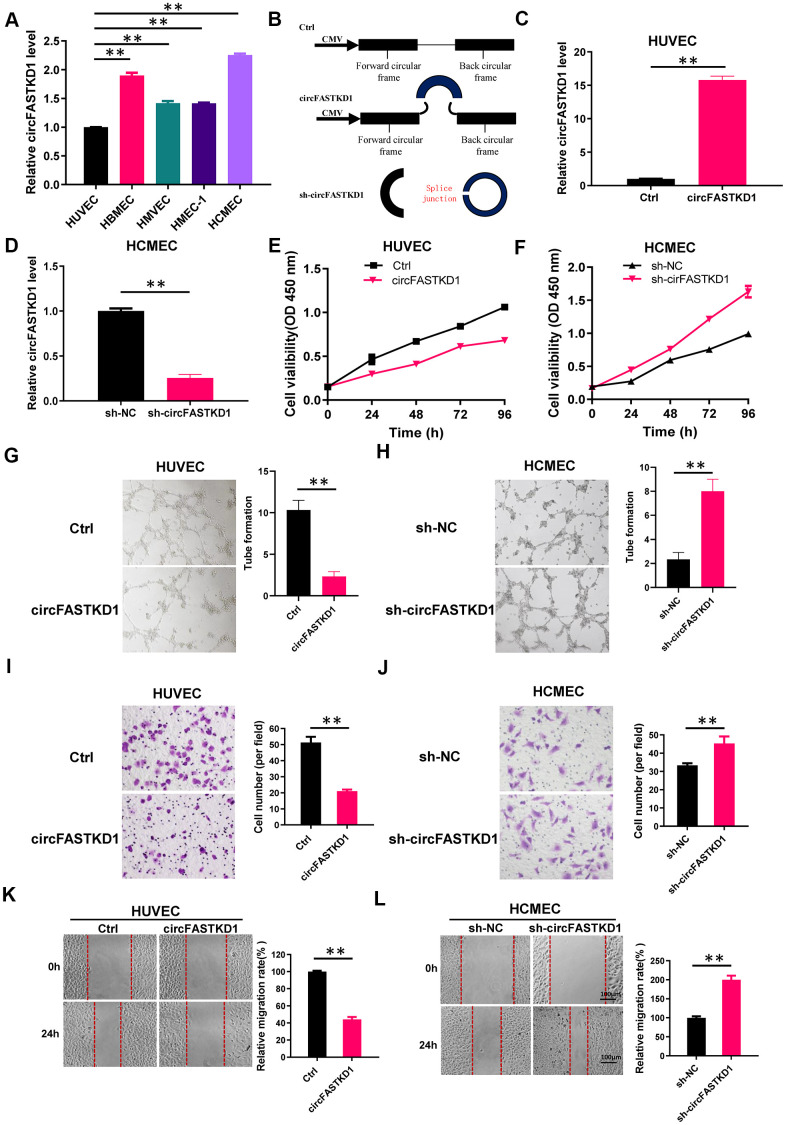
**Function of circFASTKD1 in angiogenesis *in vitro*.** (**A**) CircFASTKD1 levels were determined via qRT-PCR in HUVECs, HBMECs, HMVECs, HMEC-1 cells and HCMECs. (**B**) Structures of the control (Ctrl), circFASTKD1 and sh-circFASTKD1 vectors. (**C**, **D**) CircFASTKD1 levels were determined via qRT-PCR in HUVECs transfected with the Ctrl or circFASTKD1 vector (**C**) and in HCMECs transfected with the sh-NC or sh-circFASTKD1 vector (**D**). (**E**, **F**) The cell growth curves of HUVECs (**E**) and HCMECs (**F**) transfected with the indicated vectors were determined using a CCK-8 assay. (**G**, **H**) The effects of transfection with the indicated vectors on the tube formation abilities of HUVECs (**G**) and HCMECs (**H**). (**I**, **J**) Transwell chambers were used to perform cell migration assays in HUVECs (**I**) or HCMECs (**J**) transfected with the indicated vectors. (**K**, **L**) Wound healing assays were used to examine the motility of HUVECs (**K**) and HCMECs (**L**) transfected with the indicated vectors. Data are presented as the mean of three experiments, and the error bars represent the SD (*P<0.05 and **P<0.01).

In this study, we first assessed circFASTKD1 expression in five vascular endothelial cell lines using quantitative real-time (qRT)-PCR. CircFASTKD1 expression was the lowest in human umbilical vein endothelial cells (HUVECs) and the highest in human cardiac microvascular endothelial cells (HCMECs) (Figure 1A); thus, these cell lines were chosen for subsequent experiments. We constructed a circular transcript expression vector to overexpress circFASTKD1 in HUVECs, as well as a short hairpin RNA (shRNA) vector to silence circFASTKD1 (‘sh-circFASTKD1’) in HCMECs (Figure 1B). A qRT-PCR analysis demonstrated that the circFASTKD1 vector significantly upregulated circFASTKD1 expression compared with the control vector in HUVECs (Figure 1C), whereas the sh-circFASTKD1 vector significantly downregulated endogenous circFASTKD1 expression compared with the negative control vector (‘sh-NC’) in HCMECs (Figure 1D).

Angiogenesis is defined as new microvessel formation via branching off from existing vessels. This multistep process depends on the viability, migration, mobility and tube formation of vascular endothelial cells; thus, we assessed the effects of circFASTKD1 expression on these characteristics in our transfected cell lines. We found that the upregulation of circFASTKD1 significantly reduced the viability of HUVECs, whereas the downregulation of circFASTKD1 enhanced the viability of HCMECs (Figure 1E, 1F). A tube formation assay indicated that the angiogenic ability of the circFASTKD1-overexpressing group was relatively weak (Figure 1G, 1H). Migration and wound healing assays demonstrated that circFASTKD1 overexpression inhibited the migration and mobility of vascular endothelial cells (Figure 1I–1L). These results indicated that circFASTKD1 suppresses angiogenesis in vascular endothelial cells.

### CircFASTKD1 binds directly to miR-106a

**Figure 2:**
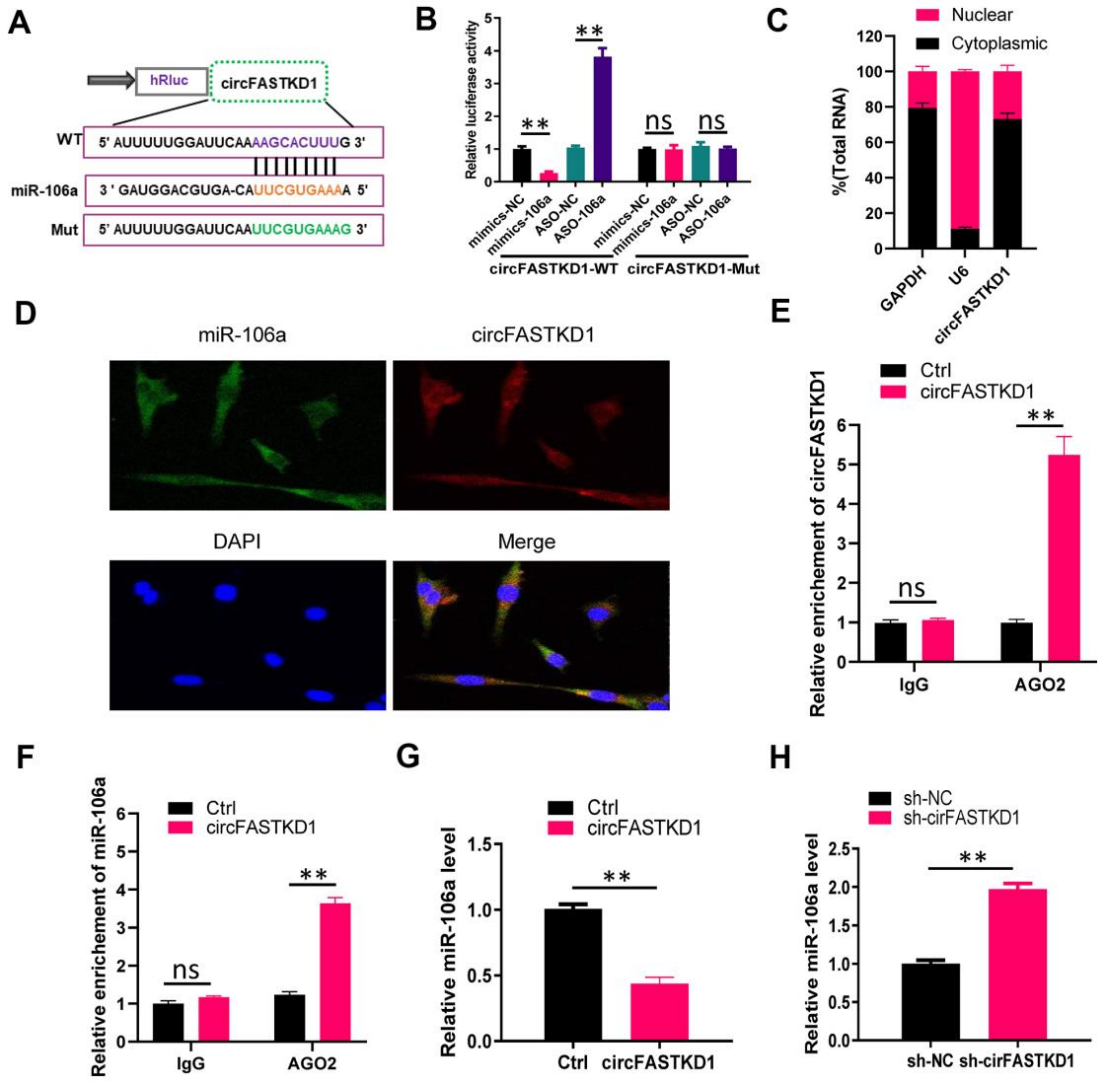
**CircFASTKD1 binds directly to miR-106a.** (**A**) Schematic diagrams of the circFASTKD1-WT and -Mut luciferase reporter vectors. (**B**) A dual-luciferase reporter assay was performed in 293T cells to verify that miR-106a was a sponged target of circFASTKD1. (**C**) CircFASTKD1 levels in the nuclear and cytoplasmic fractions of HUVECs were analyzed using qRT-PCR. (**D**) The colocalization of miR-106a with circFASTKD1 in HUVECs was detected with a FISH assay. (**E**) The association between circFASTKD1 and Ago2 was detected with a RIP assay using an Ago2 or IgG antibody. CircFASTKD1 levels in HUVECs were assessed using qRT-PCR. (**F**) The associations among circFASTKD1, miR-106a and Ago2 were detected with a RIP assay using an Ago2 or IgG antibody. MiR-106a levels in HUVECs were assessed using qRT-PCR. (**G**) MiR-106a levels were detected via qRT-PCR in HUVECs transfected with circFASTKD1 or control (Ctrl) vectors. (**H**) MiR-106a levels were detected via qRT-PCR in HCMECs transfected with sh-circFASTKD1 or sh-NC. Data are presented as the mean of three experiments, and the error bars represent the SD (*P<0.05 and **P<0.01).

Next, we used an miRNA database (Starbase) to predict miRNAs that could bind to circFASTKD1. We found that circFASTKD1 contains a sequence that complements the sequence of miR-106a. Thus, we constructed a luciferase reporter by inserting a wild-type circFASTKD1 sequence (‘circFASTKD1-WT’) or a sequence with a mutated miR-106a binding site (‘circFASTKD1-Mut’) into the 3' untranslated region (UTR) of Renilla luciferase (Figure 2A). Then, we measured luciferase activity in 293T cells co-transfected with the reporter constructs and either miR-106a mimics (‘mimics-106a’) or antisense oligos designed to inhibit miR-106a (‘ASO-106a’). The luciferase activity in circFASTKD1-WT-transfected cells was downregulated upon co-transfection with mimics-106a compared with the negative control (‘mimics-NC’), and was upregulated upon co-transfection with ASO-106a compared with the negative control (‘ASO-NC’); however, the luciferase activity did not change in circFASTKD1-Mut-transfected cells co-transfected with either mimics-106a or ASO-106a compared with their respective negative controls (Figure 2B).

To determine the cellular localization of circFASTKD1, we isolated cytoplasmic and nuclear fractions from HUVECs, with *GAPDH* and *U6* as controls, respectively. A qRT-PCR analysis demonstrated that circFASTKD1 was mostly distributed in the cytoplasmic fraction (Figure 2C). We also performed fluorescence *in situ* hybridization (FISH), which indicated that circFASTKD1 colocalized with miR-106a in HUVECs (Figure 2D).

Previous studies [20–22] have shown that miRNAs can degrade RNA or repress translation through an Ago2-dependent pathway. Thus, we performed an Ago2 RNA-binding protein immunoprecipitation (RIP) assay in HUVECs transfected with circFASTKD1. CircFASTKD1 and miR-106a were pulled down specifically in circFASTKD1 over-expressing HUVECs, indicating that circFASTKD1 directly inhibits miR-106a (Figure 2E, 2F). We also examined miR-106a expression in HUVECs transfected with circFASTKD1 and in HCMECs transfected with sh-circFASTKD1. The upregulation of circFASTKD1 dramatically inhibited the expression of miR-106a, while the downregulation of circFASTKD1 induced it (Figure 2G, 2H).

### Cell function experiments related to miR-106a

**Figure 3:**
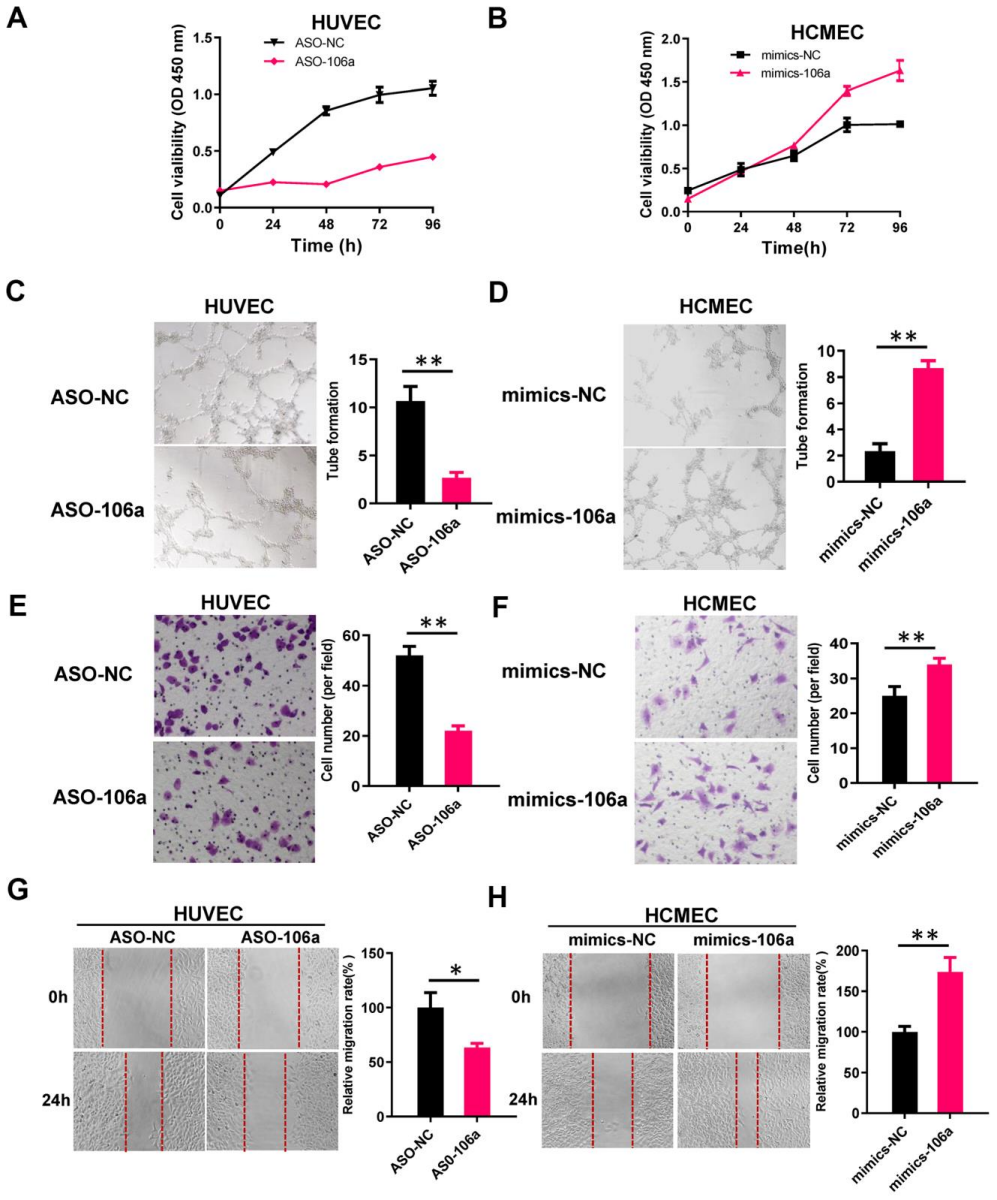
**MiR-106a enhances the angiogenic abilities of endothelial cells.** (**A**, **B**) CCK-8 assays were used to determine the growth curves of HUVECs transfected with ASO-106a or ASO-NC (**A**) and HCMECs transfected with mimics-106a or mimics-NC (**B**). (**C**, **D**) Tube formation was assessed in HUVECs transfected with ASO-106a or ASO-NC (**C**) and HCMECs transfected with mimics-106a or mimics-NC (**D**). (**E**, **F**) Transwell chambers were used to perform cell migration assays in HUVECs transfected with ASO-106a or ASO-NC (**E**) and HCMECs transfected with mimics-106a or mimics-NC (**F**). (**G**, **H**) Wound healing assays were performed to assess cell motility in HUVECs transfected with ASO-106a or ASO-NC (**G**) and HCMECs transfected with mimics-106a or mimics-NC (**H**). Data are presented as the mean of three experiments, and the error bars represent the SD (*P<0.05 and **P<0.01).

We then used a Cell Counting Kit 8 (CCK-8) assay to analyze endothelial cell growth in HUVECs transfected with ASO-106a or ASO-NC, as well as in HCMECs transfected with mimics-106a or mimics-NC. We found that miR-106a overexpression significantly enhanced the viability of endothelial cells (Figure 3A, 3B). Likewise, the ability of endothelial cells to form tube-like vascular structures was weakened in HUVECs transfected with ASO-106a, but was strengthened in HCMECs transfected with mimics-106a (Figure 3C, 3D). Cell migration and wound healing assays demonstrated that miR-106a overexpression promoted the migration and mobility of endothelial cells (Figure 3E–3H).

### MiR-106a inhibits the expression of *LATS1* and *LATS2*

**Figure 4:**
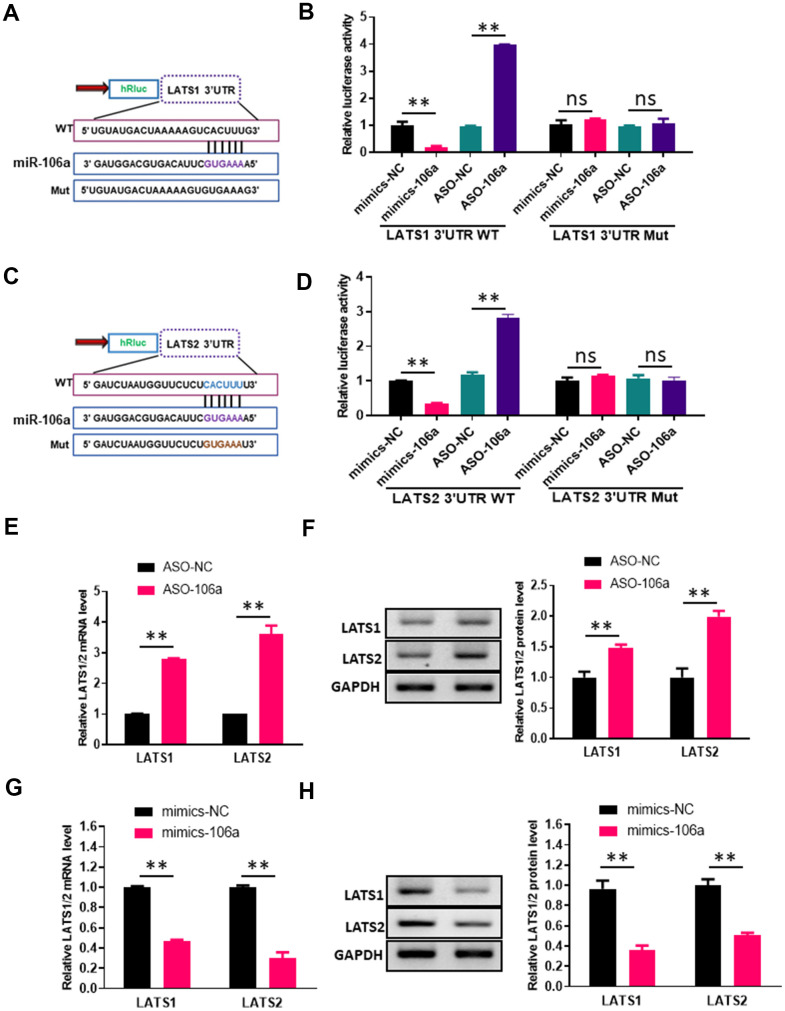
**MiR-106a directly inhibits the expression of *LATS1* and *LATS2*.** (**A**, **C**) Schematic of miR-106a and target gene prediction based on the miRBase website. (**B**, **D**) Relative luciferase activities were analyzed in 293T cells transfected with the wild-type or mutant luciferase reporter vector for the *LATS1* 3'UTR (**B**) or the *LATS2* 3'UTR (**D**) and co-transfected with mimics-106a, mimics-NC, ASO-106a or ASO-NC. (**E**, **G**) *LATS1* and *LATS2* mRNA levels were analyzed via qRT-PCR in HUVECs transfected with ASO-106a or ASO-NC (**E**) and HCMECs transfected with mimics-106a or mimics-NC (**G**). (**F**, **H**) LATS1 and LATS2 protein levels were assessed using Western blotting in HUVECs transfected with ASO-106a or ASO-NC (**F**) and HCMECs transfected with mimics-106a or mimics-NC (**H**).

According to miRBase (an miRNA database), the 3'UTRs of both large tumor suppressor kinase 1 (*LATS1*) and *LATS2* contain binding sites for miR-106a (Figure 4A, 4C). To validate this potential binding, we transfected 293T cells with luciferase reporter constructs containing either wild-type or mutated sequences from the 3'UTRs of *LATS1* or *LATS2*, and co-transfected them with either mimics-106a, ASO-106a or the corresponding negative controls. Co-transfection with mimics-106a reduced the luciferase reporter activities of the LATS1-3'UTR-WT and LATS2-3'UTR-WT constructs compared with mimics-NC, while co-transfection with ASO-106a increased the luciferase reporter activities of these constructs compared with ASO-NC (Figure 4B, 4D). However, neither mimics-106a nor ASO-106a altered the luciferase reporter activities of the LATS1-3'UTR-Mut and LATS2-3'UTR-Mut constructs.

We then performed qRT-PCR and Western blotting analyses in HUVECs transfected with ASO-106a or ASO-NC, as well as in HCMECs transfected with mimics-106a or mimics-NC. The qRT-PCR results indicated that transfection with ASO-106a increased the mRNA levels of *LATS1* and *LATS2*, while transfection with mimics-106a significantly reduced them (Figure 4E, 4G). Likewise, the Western blotting results demonstrated that the downregulation of miR-106a with ASO-106a significantly increased LATS1 and LATS2 protein levels, whereas the upregulation of miR-106a with mimics-106a significantly reduced LATS1 and LATS2 protein levels (Figure 4F, 4H). These findings indicated that miR-106a can bind to and inhibit *LATS1* and *LATS2*.

### Overexpression of miR-106a reverses the effects of circFASTKD1

**Figure 5:**
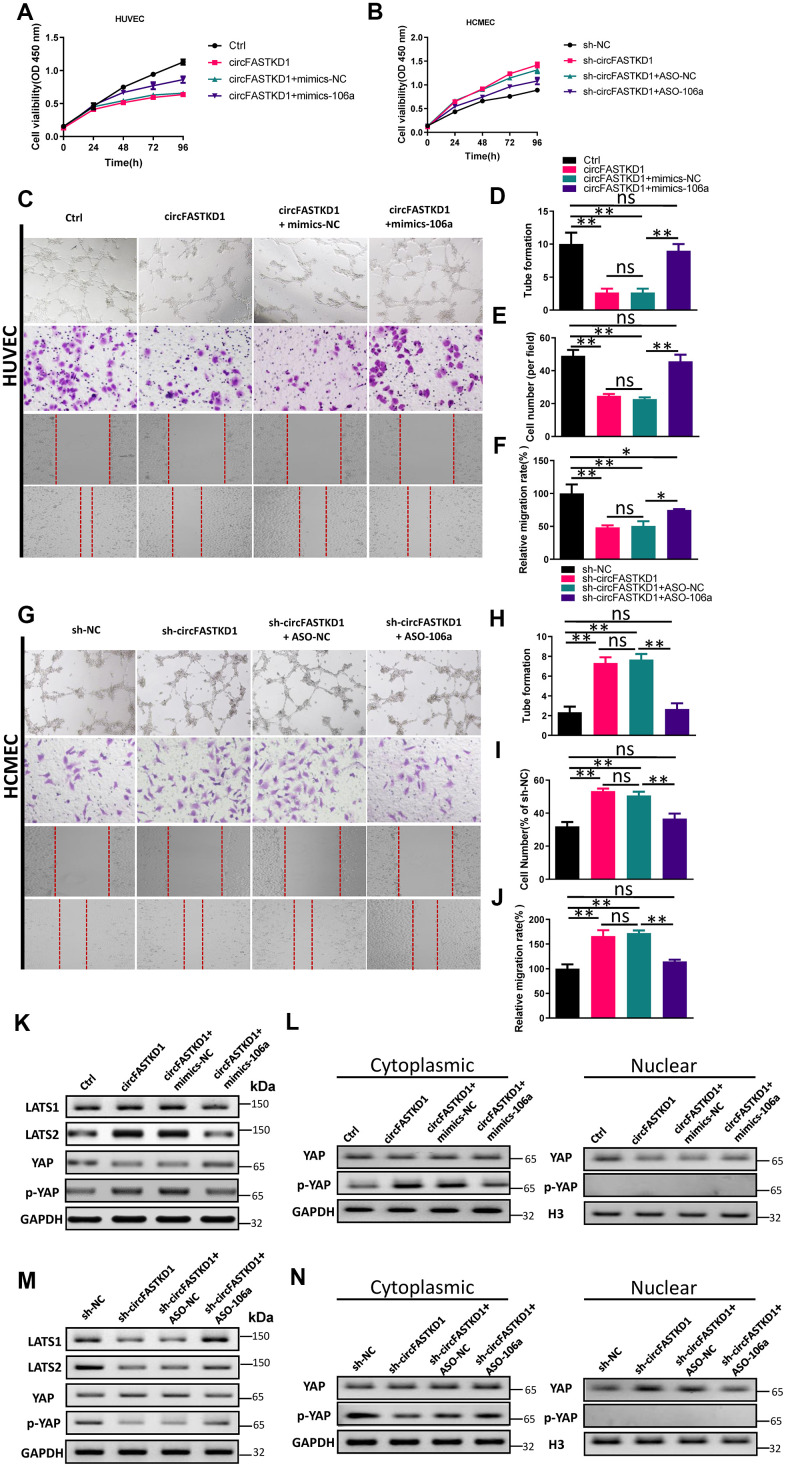
**Overexpression of miR-106a reverses the effects of circFASTKD1.** (**A**, **B**) CCK-8 assays were used to determine the growth curves of HUVECs (**A**) or HCMECs (**B**) transfected with the indicated vectors. (**C**) Photographs from the tube formation, cell migration and wound healing assays in HUVECs. (**D**–**F**) Quantitative analyses of the tube formation (**D**), cell migration (**E**) and wound healing (**F**) assays in HUVECs. (**G**) Photographs from the tube formation, cell migration and wound healing assays in HCMECs. (**H**–**J**) Quantitative analyses of the tube formation (**H**), cell migration (**I**) and wound healing (**J**) assays in HCMECs. (**K**, **M**) Western blotting analyses of LATS1, LATS2, YAP and p-YAP protein levels in HUVECs (**K**) and HCMECs (**M**) following the rescue experiments. (**L**, **N**) Western blotting analyses of the subcellular levels of YAP and p-YAP in HUVECs (**L**) and HCMECs (**N**). Data are presented as the mean of three experiments, and the error bars represent the SD (*P<0.05 and **P<0.01).

Next, we tested whether the overexpression of miR-106a could counteract the effects of circFASTKD1 on angiogenesis. Indeed, while the overexpression of circFASTKD1 reduced the viability, migration, mobility and tube-like vascular structure formation of HUVECs, the overexpression of miR-106a reversed these effects (Figure 5A, 5C–5F). Likewise, the knockdown of circFASTKD1 increased the viability, migration, mobility and tube-like vascular structure formation of HCMECs, while the inhibition of miR-106a reversed these effects (Figure 5B, 5G–5J). These results indicated that miR-106a is critical for circFASTKD1 function.

To further explore the molecular mechanism whereby circFASTKD1 inhibits angiogenesis, we measured the protein levels of LATS1, LATS2 and phosphorylated Yes-associated protein (p-YAP) using Western blotting. The results indicated that the upregulation of circFASTKD1 significantly increased LATS1, LATS2 and p-YAP levels, whereas the downregulation of circFASTKD1 reduced them. However, co-transfection of circFASTKD1-overexpressing cells with mimics-106a or co-transfection of circFASTKD1-knockdown cells with ASO-106a neutralized the effects of circFASTKD1 expression on LATS1, LATS2 and p-YAP expression (Figure 5K, 5M).

We also used Western blotting to assess the subcellular levels of p-YAP and YAP in our transfected HUVECs and HCMECs. The up- and downregulation of circFASTKD1 respectively inhibited and promoted the nuclear translocation of YAP, whereas mimics-106a and ASO-106a respectively neutralized these effects (Figure 5L, 5N). The above results demonstrated that circFASTKD1 alters the angiogenic abilities of vascular endothelial cells through the miR-106a/LATS1/2/YAP pathway.

### CircFASTKD1 downregulation enhances the angiogenic abilities of vascular endothelial cells under hypoxic conditions

To assess the function of circFASTKD1 under hypoxic conditions, we first used qRT-PCR to measure circFASTKD1 levels in HUVECs and HCMECs subjected to hypoxia for 24 h. In both cell lines, hypoxia significantly increased the expression of circFASTKD1 (Figure 6A, 6B). CCK-8 assays demonstrated that the up- and downregulation of circFASTKD1 respectively reduced and increased the viability of vascular endothelial cells under hypoxic conditions (Figure 6C, 6D). Tube formation assays demonstrated that the up- and downregulation of circFASTKD1 respectively reduced and increased the ability of vascular endothelial cells to form tubular structures during hypoxia (Figure 6E, 6F, 6I, 6J). Transwell assays revealed that the number of migrating cells significantly decreased when circFASTKD1 was upregulated and increased when circFASTKD1 was downregulated under hypoxic conditions (Figure 6E, 6G, 6I, 6K). Additionally, in wound healing assays, circFASTKD1 overexpression reduced cell mobility during hypoxia, whereas circFASTKD1 downregulation increased it (Figure 6E, 6H, 6I, 6L). These results indicated that the downregulation of circFASTKD1 enhances angiogenesis in HUVEC and HCMECs.

**Figure 6:**
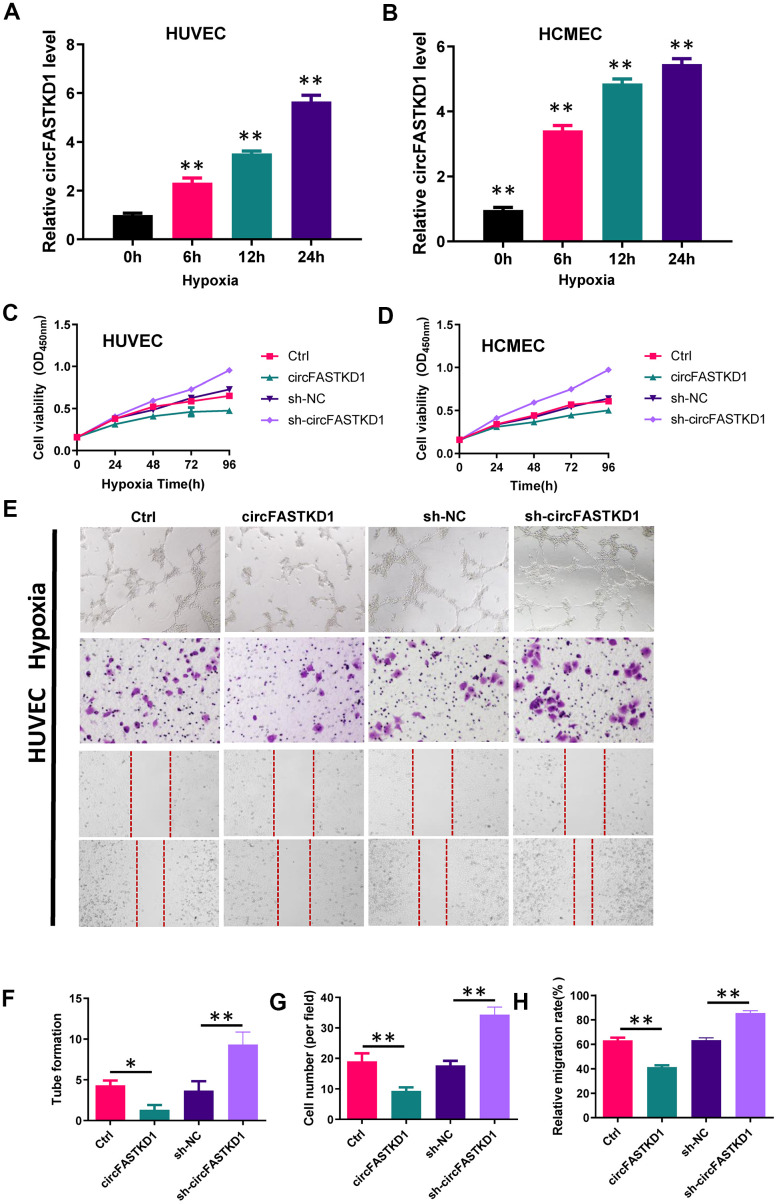
**The downregulation of circFASTKD1 benefits vascular endothelial cells under hypoxic conditions.** (**A**, **B**) qRT-PCR was used to determine circFASTKD1 levels in HUVECs (**A**) and HCMECs (**B**) under hypoxic conditions. (**C**, **D**) CCK-8 assays were used to determine the growth curves of HUVECs (**C**) and HCMECs (**D**) with up/downregulated circFASTKD1 under hypoxic conditions. (**E**) Photographs from the tube formation, cell migration and wound healing assays in HUVECs with different treatments under hypoxic conditions. (**F**–**H**) Quantitative analyses of the tube formation (**F**), cell migration (**G**) and wound healing (**H**) assays in HUVECs under hypoxic conditions.

**Figure 6:**
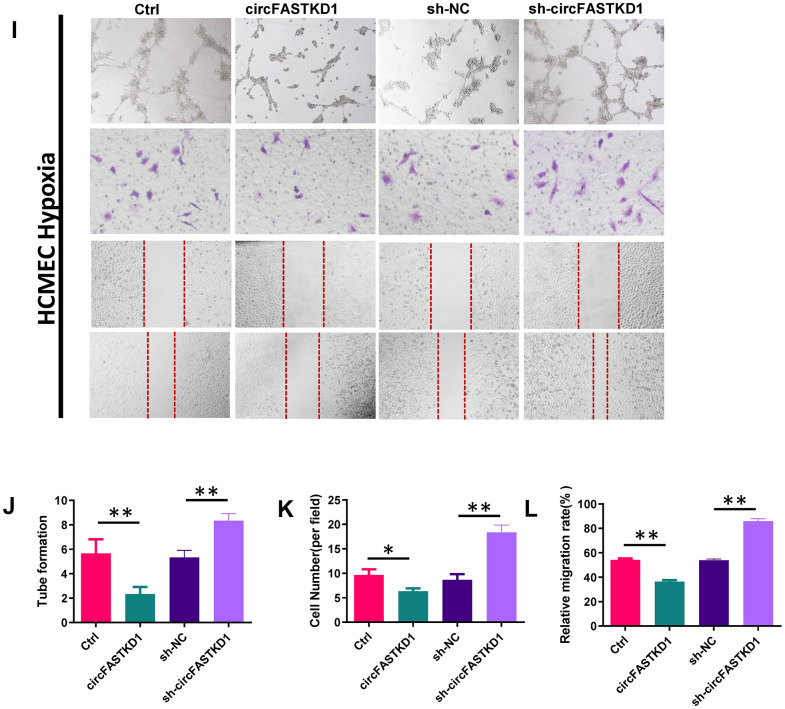
**The downregulation of circFASTKD1 benefits vascular endothelial cells under hypoxic conditions.** (**I**) Photographs from the tube formation, cell migration and wound healing assays in HCMECs with different treatments under hypoxic conditions. (**J**–**L**) Quantitative analyses of the tube formation (**J**), cell migration (**K**) and wound healing (**L**) assays in HCMECs under hypoxic conditions.

### CircFASTKD1 downregulation ameliorates myocardial infarction

**Figure 7:**
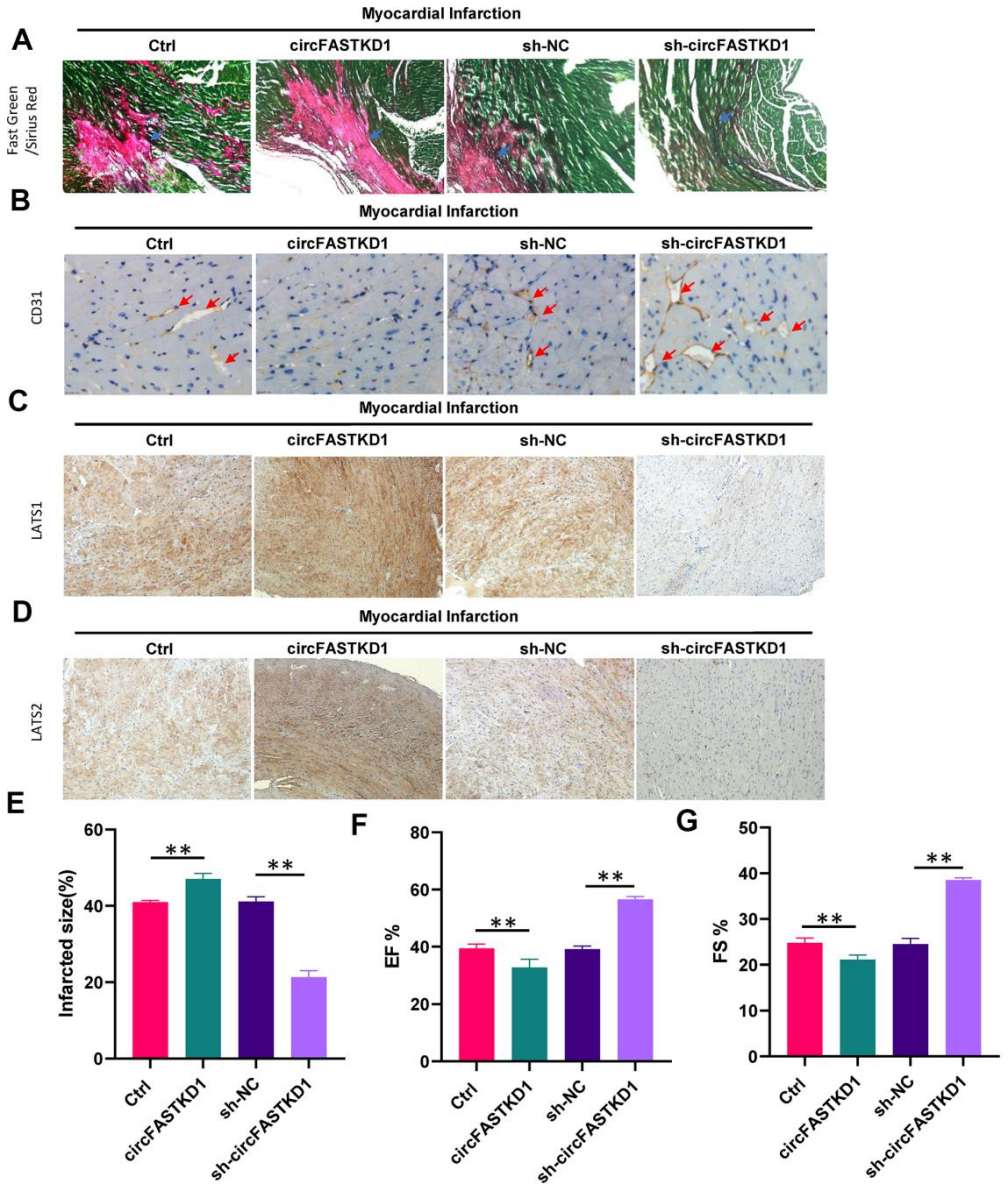
**The downregulation of circFASTKD1 ameliorates myocardial infarction.** (**A**) Fast Green and Sirius Red staining to mark the myocardium (green) and scar (red) in each group. (**B**) CD31 staining showing the microvessel density in each group. (**C**, **D**) Immunohistochemical staining showing the expression of LATS1 (**C**) and LATS2 (**D**) in myocardial tissue from the different groups. (**E**–**G**) Quantification of the scar size (**E**), left ventricular EF% (**F**) and left ventricular FS% (**G**) in the different groups. Data are presented as the mean of three experiments, and the error bars represent the SD (*P<0.05 and **P<0.01).

Finally, we conducted *in vivo* experiments to determine the impact of circFASTKD1 expression on myocardial ischemia. Mice were subjected to myocardial infarction, and then were injected twice a week for four weeks with lentiviral circFASTKD1, sh-circFASTKD1 or negative control vectors. The proportion of necrotic fibrous tissue in the heart following myocardial infarction was elevated in the circFASTKD1 group, but was reduced in the sh-circFASTKD1 group (Figure 7A, 7E). CD31 staining revealed that the microvessel density was reduced in the circFASTKD1 group, but was elevated in the sh-circFASTKD1 group (Figure 7B). Immunohistochemical analyses indicated that the up- and downregulation of circFASTKD1 respectively promoted and inhibited the expression of LATS1 and LATS2 in myocardial tissue (Figure 7C, 7D). In terms of cardiac function, the ejection fraction (EF) and fractional shortening (FS) were significantly better in the sh-circFASTKD1 group than in the sh-NC group (Figure 7F, 7G). These results suggested that the downregulation of circFASTKD1 ameliorates myocardial infarction.

## DISCUSSION

The abundance of circRNAs in eukaryotes was only recognized very recently, and few circRNAs have been well-characterized and functionally demonstrated to be miRNA sponges/inhibitors. In this work, we identified circFASTKD1 as a new angiogenesis-related circRNA. In terms of its function, circFASTKD1 was found to suppress the angiogenesis of vascular endothelial cells. In terms of its mechanism, circFASTKD1 operated as a complementary endogenous RNA to miR-106a, thus de-repressing the miR-106a target genes *LATS1* and *LATS2* and inhibiting the YAP signaling pathway during vascular endothelial progression. Overall, our data suggest that circFASTKD1 may be an important inhibitor of angiogenesis in vascular endothelial cells.

Hippo/YAP signal transduction is an evolutionarily conserved pathway that has been characterized as a key determinant of cell viability, tumorigenesis and metastasis [23–26]. LATS1 and LATS2, the upstream serine/threonine kinases of YAP in the classical Hippo/YAP pathway, are crucial in the cell cycle because they reduce cell viability, inhibit cell migration and maintain cell homeostasis [27–29]. The downregulation of LATS1 and LATS2 has been observed in astrocytoma, breast cancer, colon cancer, gliomas and non-small cell lung cancer, and is directly associated with a poor prognosis [30–32]. However, the functions of LATS1 and LATS2 in the vascular endothelium have been unclear thus far. Our study revealed that the upregulation of circFASTKD1 promoted LATS1 and LATS2 expression, thus inhibiting the YAP signaling pathway and suppressing vascular endothelial angiogenesis *in vitro*. CircFASTKD1 functioned as an endogenous sponge for miR-106a in endothelial cells, and miR-106a was identified as an inhibitor of *LATS1* and *LATS2*. In fact, we found identical miR-106a response elements in circFASTKD1 and the 3'UTRs of *LATS1* and *LATS2*. Our qRT-PCR and luciferase reporter assays suggested that circFASTKD1 binds competitively to miR-106a, thus preventing it from binding to *LATS1* and *LATS2*. Therefore, circFASTKD1 induces *LATS1* and *LATS2* expression by antagonizing miR-106a.

Hypoxia is a key angiogenic factor. To investigate the involvement of circFASTKD1 in myocardial infarction, we simulated myocardial infarction *in vitro* by subjecting vascular endothelial cells to hypoxia. CircFASTKD1 was significantly upregulated in HUVECs and HCMECs under hypoxic conditions. The silencing of circFASTKD1 enhanced the viability, migration and angiogenesis of vascular endothelial cells during hypoxia. These results implied that the downregulation of circFASTKD1 could ameliorate myocardial infarction by promoting angiogenesis.

We also generated a mouse model of myocardial infarction, and confirmed that it was successfully established based on the observed infarct area. We then used this model to explore the effects of circFASTKD1 inhibition *in vivo*. After 28 days, the downregulation of circFASTKD1 reduced the degree of myocardial tissue damage, diminished the proportion of myocardial necrotic fibrous tissue, inhibited the expression of LATS1 and LATS2, and increased the EF and FS. Thus, the inhibition of circFASTKD1 improved the cardiac function of mice after myocardial infarction. Our study is the first to demonstrate the existence and functional significance of the circFASTKD1/miR-106a/LATS1/2 axis in hypoxia-stimulated vascular endothelial cells. The protective effects of downregulating circFASTKD1 following myocardial infarction can be explained by our findings that circFASTKD1 sponges miR-106a and thus de-represses *LATS1* and *LATS2*.

**Figure 8:**
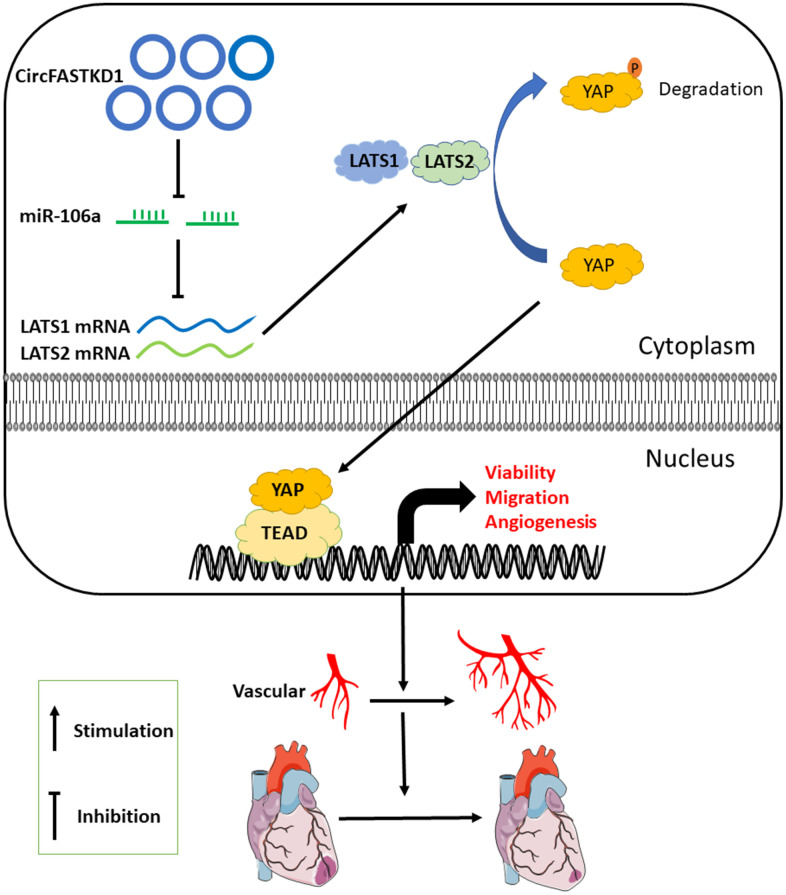
**Proposed mechanism.** This investigation has suggested that, by binding to miR-106a, circFASTKD1 induces LATS1 and LATS2 expression and inhibits the YAP pathway, thus suppressing angiogenesis, increasing the infarct size and inhibiting repair in the heart following myocardial infarction.

In conclusion, circFASTKD1 is a candidate suppressor of angiogenesis in endothelial cells. By sponging miR-106a, circFASTKD1 disinhibits *LATS1* and *LATS2,* thus inhibiting the YAP signaling pathway and suppressing endothelial cell growth, migration, mobility and angiogenesis. The downregulation of circFASTKD1 ameliorates myocardial infarction by promoting angiogenesis through the miR-106a/LATS1/2/YAP pathway *in vitro* and *in vivo*, as indicated in Figure 8. Our results suggest that circFASTKD1 exerts important physiological effects in cardiovascular diseases and could be a new prognostic biomarker and therapeutic target in myocardial infarction patients.

## MATERIALS AND METHODS

### Cell culture

HUVECs, HCMECs and an immortalized human dermal microvascular endothelial cell line (HMEC-1) were purchased from BN Biotech (Beijing, China). A human brain microvascular endothelial cell line (HBMEC) and a human microvascular endothelial cell line (HMVEC) were purchased from Fenghui Biotech (Changsha, China). The cells were cultured in RPMI-1640 medium (Hyclone, USA) supplemented with 10% fetal bovine serum (Hyclone) and 1% penicillin-streptomycin in a humidified atmosphere containing 5% CO_2_ at 37° C. For the hypoxia experiments, the cells were seeded in an atmosphere containing 1% O_2_, 5% CO_2_ and 94% N_2_ for 24 h in a hypoxic chamber.

### CCK-8 assay

Cell viability was determined with a CCK-8 assay in accordance with the manufacturer’s instructions. Cells were seeded on 96-well plates at 2×10^3^ cells/well. The cells were transfected 24 h after being seeded, and were then cultivated for 24, 48, 72 or 96 h. Subsequently, the medium was replaced with 100 μL of RPMI-1640 medium, and 10 μL of CCK-8 reagent was added. The cells were then incubated for 3 h at 37° C, and the optical density was measured with a Multiskan™ FC (Thermo Scientific, USA) at 450 nm.

### Cell migration assay

Cell migration was assessed through Transwell assays. HUVECs and HCMECs with different treatments were added to the top-chamber inserts of Transwell assay plates (BD Biosciences, USA), while the bottom of each chamber was filled with 600 μL of medium containing 10% fetal bovine serum. After being cultured at 37° C for 24 h, the cells that had migrated through the filter membrane were washed three times with 1× phosphate-buffered saline (PBS), fixed in 4% paraformaldehyde (pre-cooled at 4° C) and stained with a crystal violet staining solution (KeyGEN BioTECH, China). The cells that had migrated were then counted under a microscope (Nikon, Japan).

### Wound healing assay

Cell mobility was assessed through wound healing assays. Cells were seeded on 35-mm dishes to form 100% confluent monolayers. The cells were wounded with 100-μL tips, washed with PBS and then subjected to different treatments. Wound images were obtained at 0 and 24 h with a Nikon microscope (40× magnification), and the wound healing speed was calculated.

### qRT-PCR

Total RNA was extracted with Trizol reagent (Invitrogen, USA) and then reverse-transcribed into cDNA. Reverse transcription was performed with PrimeScript™ RT Master Mix (TaKaRa, Japan), and cDNA amplification was conducted using SYBR Green Premix Ex Taq™ II (TaKaRa) in accordance with the manufacturer’s instructions. *U6* was used for miR-106a template normalization, while *GAPDH* was used for circFASTKD1 and coding gene template normalization. The 2^-ΔΔCT^ method was used to calculate the relative gene levels.

### Plasmids, shRNAs, miRNA mimics and ASOs

We obtained a circFASTKD1 overexpression plasmid and a circFASTKD1 shRNA vector, and used an empty vector as a control. The circFASTKD1 shRNA sequence was 5'-GTCTCAATTAAATCCATCCTT-3'. MiRNA mimics and ASOs were purchased from OBIO Co., Ltd (Shanghai, China). The mimics-106a sequence was 5'-AAAAGUGCUUACAGUGCAGGUAG-3'. The mimics-NC sequence was 5'-UUCUCCGAACGUGUCACGUTT-3'. The ASO-106a sequence was 5'-CUACCUGCACUGUAAGCACUUUU-3'. The ASO-NC sequence was 5'-CAGUACUUUUGUGUAGUACAA-3'. HUVECs and HCMECs were plated at 50-60% confluence on six-well plates 24 h prior to transfection. The cells were then transfected using Lipofectamine 2000 (Invitrogen) in accordance with the manufacturer’s instructions.

### Tube formation assay

Matrigel was thawed on ice and plated on 96-well plates (100 μL per well), which were then placed in a 37° C incubator for 30 min to allow a gel to form. Approximately 2×10^4^ cells were added per well. The endothelial tube was examined under a microscope every 5 h, and the numbers of branches and nodules in the formed tube were evaluated.

### Western blot analysis

HUVECs and HCMECs were incubated overnight at a density of 3×10^5^ cells/well on a six-well plate. After being subjected to different treatments, the cells were collected and lysed in radioimmunoprecipitation assay lysis buffer containing a protease inhibitor (Selleck, USA) on ice. The lysate proteins were quantified, and 25 μg from each sample was separated by sodium dodecyl sulfate polyacrylamide gel electrophoresis and transferred to a polyvinylidene difluoride membrane. Nonspecific binding sites were blocked with 5% nonfat milk, and the membrane was incubated with primary antibodies at 4° C overnight. The proteins were visualized with enhanced chemiluminescence substrate reagents (Millipore, USA). The primary antibodies used in this study included GAPDH (Saierbio, China), Histone H3 (Bioss, China), LATS1 (Bioss), LATS2 (Bioss), YAP (Affinity, China) and p-YAP (Affinity). A horseradish peroxidase-conjugated goat anti-rabbit (Affinity) or anti-mouse IgG antibody (Affinity) was used as the secondary antibody. GAPDH was used as a loading control. Histone H3 was used as a loading control for nuclear protein.

### Dual-luciferase reporter assay

Dual-luciferase reporter constructs were transfected into 293T cells using transfection reagents. Twenty-four hours after the transfection, the medium was replaced with fresh medium and the cells were treated in different ways. The culture medium was transferred to a 96-well white plate after 48 h, and luminescence was measured with a luminometer.

### Subcellular distribution

Cytoplasmic and nuclear RNA was extracted with a PARIS Kit (Life Technologies, USA). The total RNA in each fraction was quantified using qRT-RCR. *GAPDH* and *U6* were used as internal references for the cytoplasm and nucleus, respectively.

### RIP assay

Cells were washed with ice-cold PBS, scraped off their dishes using a cell lifter and centrifuged at 1500 rpm for 5 min. The cell pellet was collected and resuspended in a lysis buffer containing 150 mM potassium chloride, 25 mM Tris-HCl (pH 7.4), 5 mM ethylenediaminetetraacetic acid, 0.5% Triton X-100 and 5 mM dithiothreitol supplemented with an RNase inhibitor (TaKaRa) and a proteinase inhibitor cocktail (Roche, Switzerland). The suspension was incubated on ice for 5 min and centrifuged at 14,000 rpm for 5 min. Then, the lysate was incubated for 3 h at 4° C on a rotating wheel with protein A/G magnetic beads that had been pre-hybridized with an Ago2 antibody. The beads were collected and purified using protease K, and the RNA in the supernatant was isolated using Trizol reagent (Invitrogen). PCR was used to detect the enriched RNAs. Normal IgG was used as a negative control.

### FISH

An RNA-FISH assay was performed with a FISH Kit (RiboBio, Guangzhou, China). Briefly, HUVECs were fixed in 4% formaldehyde at room temperature for 10 min, washed with PBS and permeabilized with 0.5% Triton X-100 in PBS at 4° C for 30 min. Then, the cells were pre-hybridized at 37° C for 30 min and hybridized using a circRNA probe in hybridization solution at 37° C overnight. The probe sequence covered the specific junction region of circFASTKD1. After hybridization, the cells were washed with prewarmed wash buffer and PBS six times. Finally, the cells were counter-stained with 4',6-diamidino-2-phenylindole and visualized using a confocal laser scanning microscope (FV10i; Olympus, Japan).

### Animal experiments

Eight-week-old male C57BL/6 mice were used for *in vivo* experiments. All experiments were performed according to protocols approved by the Animal Ethical and Welfare Committee (Approval NO. NKYY-DWLL-2020-146). Each mouse underwent tracheal intubation and was anesthetized with isoflurane (3% for induction and 2% for maintenance). Then, the chest was opened and the left anterior descending coronary artery was ligated with 7-0 prolene sutures under aseptic conditions. Mice receiving the sham operation were subjected to the same surgical procedures, but the left anterior descending coronary artery was not ligated. After the completion of the surgery, the chest was closed and the mouse was warmed for several minutes until recovery. Subsequently, the tail vein was injected with 200ul circFASTKD1 lentivirus, sh-circFASTKD1 lentivirus or negative control lentivirus (1×10^7^ plaque-forming units/mL) twice a week for four weeks. The lentiviruses were synthesized by OBIO Co., Ltd.

### Echocardiography

On day 28 after myocardial infarction, the mice were anesthetized with isoflurane (2.5% isoflurane for induction and 0.5% for maintenance). Then, echocardiography was performed on a Visual Sonics Vevo® 2100 Imaging System (Visual Sonics, Toronto, Canada) with a 40-MHz MicroScan transducer (model MS-550D) to measure cardiac function. Functional parameters such as the FS and EF were calculated using the accompanying software.

### Histology assays

Mouse hearts were removed, rinsed and arrested in diastole using a buffer containing 4.7 nM potassium chloride and 0.1% 2,3-butanedione monoxime in PBS. The hearts were then fixed in 4% paraformaldehyde (pH 7.4) overnight. After being dehydrated through a series of ethanol baths, the samples were embedded in paraffin wax according to standard laboratory procedures. Then, 4-μm-thick sections were stained with hematoxylin and eosin and subjected to routine histological examination under a light microscope.

Immunohistochemical staining was used to determine LATS1 and LATS2 levels in myocardial tissue. Sections of 4 μm were dehydrated, heated in citric acid buffer for antigen repair, and stained with specific antibodies against LATS1 and LATS2 (Bioss).

For the determination of the infarct size, the hearts were fixed in 4% paraformaldehyde (pH 8.0) before being dehydrated and embedded in paraffin. Then, the embedded paraffin blocks were sectioned from the apex to the base, and 10-μm-thick sections were stained with Fast Green and Sirius Red.

### Statistical analysis

Statistical analyses were performed with SPSS 24.0 (SPSS, Inc., Chicago, IL, USA) and GraphPad Prism 7.0 (GraphPad Software, USA). The data are presented as the mean ± standard deviation (SD). The groups were compared using two-tailed unpaired Student’s *t* tests or one-way analysis of variance. P-values less than 0.05 were considered significant.
